# Perspective: The Tailgate Medicine Model: A Proposed Integrative Method of Care Delivery for Rural Populations

**DOI:** 10.1002/hsr2.71959

**Published:** 2026-03-18

**Authors:** Michael P. McShane, Jennifer A. Kowalkowski, Kristina Brant, Joel Segel, Ashley Visco, Philip Volpe, Mark Stephens

**Affiliations:** ^1^ Department of General Internal Medicine Penn State College of Medicine, Penn State University State College Pennsylvania USA; ^2^ Ross and Carol Nese College of Nursing Penn State University State College Pennsylvania USA; ^3^ Department of Agricultural Economics, Sociology, and Education College of Agricultural Sciences, Penn State University State College Pennsylvania USA; ^4^ Consortium on Substance Use and Addiction, Social Science Research Institute Penn State University State College Pennsylvania USA; ^5^ Department of Health Policy and Administration College of Health and Human Development, Penn State University State College Pennsylvania USA; ^6^ Department of Public Health Sciences Penn State College of Medicine Hershey Pennsylvania USA; ^7^ EDI Shared Services University of Maryland Medical System Baltimore Maryland USA; ^8^ Department of Family Medicine Kansas City University of Medicine and Biosciences Kansas City Missouri USA; ^9^ Department of Family and Community Medicine Penn State College of Medicine, Penn State University State College Pennsylvania USA

**Keywords:** delivery of care, health services accessibility, mobile health, rural health, social determinants of health

## Abstract

**Background and Aims:**

Social, environmental, economic, and cultural factors—social determinants of health (SDOH)—create structural barriers that limit access to health resources and negatively impact health outcomes, particularly for rural populations. Given the significant impact of SDOH on health and mortality, there is an urgent need to develop rural care delivery models that consider community‐specific SDOH that currently drive structural inequities in health for rural populations.

**Objective:**

To propose and describe the Tailgate Medicine Model (TMM), an innovative health care delivery model that, by design, emphasizes assessment and impact of SDOH on health outcomes for rural populations. TMM blends existing strengths of both mobile health and street medicine clinics, with strategies adapted from the United States military to provide rapid medical treatment in field settings. The ability to reach underserved communities or groups, create bridges to existing health resources and social services, and scale to meet local needs make the TMM particularly well suited for rural contexts.

**Conclusion:**

Existing care delivery models fail to address many of the structural factors that perpetuate health inequities in rural communities. Despite being untested in the community, the highly adaptable TMM has potential to directly address SDOH, and holds promise for improving access to care, sharing available resources and reducing health disparities–particularly in rural settings.

## Summary

- Current health care delivery models fail to address structural inequities that perpetuate rural health disparities.
- Models are needed to address social determinants of health in a way that is tailored to local contexts and easily scalable.
- The proposed Tailgate Medicine Model leverages strengths of existing care delivery models to meet the unique health needs of rural communities.

## 1 Introduction

Healthcare delivery models define how health services are organized and delivered. Whether embedded within health care systems or stand‐alone providers, these models significantly influence quality and health outcomes, as well as health equity. Contemporary care delivery models have evolved in response to numerous factors, including costs of providing care, provider availability, demographic shifts (e.g., aging populations) and health care needs (e.g., chronic disease management). At the same time, alternative models of health care delivery have emerged to meet the needs of populations that are typically not served by existing health systems. These often include communities or groups lacking access to care and who experience notable disparities in health. Mobile health clinics (MHCs) and Street Medicine (SM) are two such care delivery models in the United States (US) focused on health equity and designed to increase access to health services, improve health outcomes, and reduce costs for underserved and vulnerable populations [1, 2, 3].

Both MHCs and SM models deliver care outside of traditional “brick and mortar” health care settings by bringing services to people within their lived environment [2, 4, 5]. This allows providers to more directly observe many important social determinants of health (SDOH) Guerra et al. [6]. MHCs have been used to address health needs in communities where transportation and geography limit access to care, such as rural and remote areas [7]. Conversely, SM was created to bring care to persons experiencing homelessness, typically in urban areas [3]. Both models, by design, emphasize the importance of building and maintaining trusting relationships with communities to reduce the incidence of delayed or deferred care, improve self‐management of chronic conditions, address health disparities, and overcome structural barriers to care [1, 3, 8, 9]. Despite many successes, inherent limitations of each model reduce their effectiveness for broad implementation in rural settings. For example, local infrastructure may not support the spatial requirements of MHCs, which are typically large mobile units. In addition, geographic spread of populations in rural areas often limits the effectiveness and efficiency of SM to deliver care on foot. Geographic and infrastructure factors limit the adaptability and utility of both MHCs and SM to address SDOH and other factors that contribute to structural inequities in rural areas.

We propose a new care delivery model, the Tailgate Medicine Model (TMM), that is conceptually grounded in an approach used by the US military to deliver care in resource‐limited geographic areas. TMM specifically leverages key strengths of both MHC and SM to: 1) better address the unique health needs of communities in rural and remote locations, 2) directly visualize, access, and intervene upon SDOH factors that drive health inequities, and 3) create bridges to integrate people with health needs into existing health and social systems. We believe the TMM offers a unique and important patient‐centered approach to improve access to care, reduce health inequities and elevate health equity for rural populations.

### 1.1 Health Challenges in Rural and Remote Settings

Geographic health disparities, differences in health status, access to health care, and health outcomes by geographic location, are pressing concerns both in the U.S. and globally [10]. People in rural and remote areas have fewer health care providers, greater travel distances to care settings, lower rates of insurance, and higher rates of both chronic disease and mortality from chronic disease [10, 11, 12]. In the U.S, more than 60% of all areas identified with shortages of primary care [13] dental, and mental health professionals are rural [14]. Living in rural areas is also associated with a greater travel time burden to obtain health care services. One study showed that rural residents travel two to three times farther to access health services than their urban counterparts [15], and another reported that rural residents spent more time traveling for health care services than for work [16]. Health system consolidation and local clinic closures in the U.S. have contributed to increasing travel distances in recent years [17, 18]. Rural residents both in the U.S. and globally are less likely to have health insurance or similar health services coverage. In the U.S. approximately 12% of people in urban areas lack health insurance while 14% of rural residents lack coverage [19, 20]. Moreover, a greater share of rural residents is covered by public insurance (45.2%), including Medicare and Medicaid, than urban residents (36.1%) [21]. Therefore, it is not surprising that rural residents experience higher rates of mortality from chronic illness. For example, in the U.S. rates of death per 100,000 population for the five leading causes of death are higher for those living in rural areas than those living in urban areas. This includes heart disease (rural 189.1, urban 156.3), cancer (rural 164.2, urban 142.8), chronic lower respiratory diseases (rural 52.5, urban 35.4), stroke (rural 39.0, urban 36.6), and unintentional injury (rural 61.1, urban 47.4) [14, 22]. These inequities are rooted in and influenced by socioeconomic, political, and environmental factors and reinforced by SDOH [10, 12]. Although specific drivers of rural‐urban health disparities and their impacts can vary, similarities are seen across the globe.

### 1.2 Conceptual Origins of Tailgate Medicine

The US military developed the concept of tailgate medicine during the Iraq and Afghanistan conflicts as an approach to provide rapid medical treatment in the field [13, 23, 24, 25]. To reduce delays in care, medical personnel embedded with forward deployed US forces ‘dropped the tailgate' of a support vehicle to set up an aid station for triage and immediate care for military personnel and local nationals in the area of operations [26]. In many of these areas, local health care infrastructure was lacking and the tailgate medicine approach filled crucial health care needs [27]. Offering services within local communities also fostered goodwill and improved social relations between military operators and local residents [26]. While the original intent of this approach was never to establish long‐term health care relationships, the use of small teams and support vehicles to deliver care and meet critical health needs in austere environments has broader application, particularly in resource‐limited geographical areas.

### 1.3 The Tailgate Medicine Model

**Figure 1:**
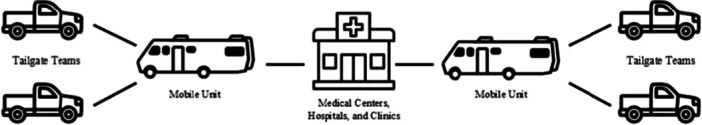
Hub‐and‐Spoke Tailgate Medicine Model. 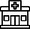 Medical centers, hospitals, and clinics serve as the central hub for more advanced services identified through mobile units and tailgate teams. 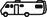 Mobile units are directly connected via primary spokes to the central hubs and bring clinical services to underserved rural communities and groups. 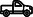 Tailgate teams are connected to mobile units via secondary spokes and use smaller vehicles to reach isolated or difficult‐to‐reach individuals or areas.

The TMM is conceptualized as a multi‐level hub‐and‐spoke care delivery model with three main components: (a) medical centers, (b) mobile units, and (c) tailgate teams (Figure 1). Medical centers are existing hospitals, clinics, or academic centers that serve as the central, or main hub from which the mobile units and tailgate teams operate. The mobile units act as direct spokes from the main hub, bringing primary or specialty care to central locations within rural communities using RVs or other vehicles that have sufficient space. These units travel to rural communities that lack local health care services and set up in a central location where they can offer primary or specialty care based on the needs of that community. The TMM is designed for a range of services, including preventive health services and chronic disease management. Mobile units can be customized to offer various laboratory services, with the types of diagnostics and treatments based on demand and expanded as technology advances. Tailgate teams act as direct spokes from the mobile units and secondary spokes from the main hub. Providers or small teams use support vehicles (e.g. cars/trucks) to deliver care in people's homes or in more remote locations that are not accessible by mobile units.

The hub‐and‐spoke design is central to the TMM's effectiveness. It creates a multi‐level system that builds bridges between different levels of care. The purpose of each component is to both extend and connect individuals and groups to the services provided by the other components, including local health systems, as well as to existing local resources. Unlike stand‐alone MHCs, the TMM creates the infrastructure to allow connections between different levels of care. For example, a community member with diabetes who is found to have high blood sugar by providers from the mobile unit can be followed up at home by a tailgate team, connected to clinic‐based diabetes educators or nutritionists, and assisted with access to specialty care including vision screening or renal care if their disease progresses. Through this multi‐level hub‐and‐spoke design, the TMM can increase access to health care services and resources for those living in rural or remote areas. This design facilitates access to and communication across the continuum of care, reducing fragmented health care delivery.

The TMM strategically integrates key elements of MHCs and SM to enhance flexibility and adaptability for the diverse needs of different communities. To begin, the TMM is designed to assure needed clinical care and social services are available to a greater proportion of residents in rural communities than MHCs or SM alone. As primary spokes from medical centers, mobile units, like MHCs, can span long distances to reach rural and remote locations that lack access to brick‐and‐mortar clinical services. However, as with other MHCs, mobile units offer services in a central location in these rural communities, still creating the potential to exclude the most vulnerable and at‐risk people. Tailgate teams, operating as secondary spokes from the mobile units can, like SM, directly address this limitation by serving residents who are even more geographically isolated or lack transportation [25].

The TMM also increases entry points to care and facilitates direct observation of SDOH, particularly among those who are uninsured, underinsured, or are underserved. For many underserved groups, entering a traditional clinic can be daunting, with the fear of doing so creating a significant barrier to care [2]. While MHCs offer more accessibility and approachability than traditional clinics [2], the person‐to‐person approach of tailgate teams, similar to SM, can provide an even lower threshold for care initiation [28] and allows for more direct observation of the impact of SDOH on health outcomes. Providers on the mobile units and in tailgate teams can visualize how SDOH play out for rural populations in ways that are not possible through care provided in brick‐and‐mortar clinical settings (e.g., lack of food, lack of heating or air conditioning, or broken stairs). Furthermore, this important information can be communicated to all providers and care teams across the TMM—from tailgate teams to the mobile unit and to medical centers—to improve appropriateness, efficiency, and effectiveness of care. By providing entry points to care and opportunities for direct observation of SDOH, the TMM can create avenues for referrals to community resources and more advanced care that can improve health outcomes and promote health equity.

The multi‐level hub‐and‐spoke design of the TMM facilitates referral to local health‐supporting resources and specialty health services. For example, an important element of MHCs and SM is their focus on connecting users to local resources, such as food pantries, housing assistance, or other social services that are essential for achieving optimal health. In addition, MHCs and SM facilitate referrals for individuals who require specialty or higher levels of care, like cardiac or diabetes specialists, imaging, or cancer treatments. These elements are incorporated into the TMM. Through its multi‐level hub‐and‐spoke design, the TMM integrates important elements of MHCs and SM which makes it a promising care delivery model for underserved rural populations.

With its goals of expanding access to care and efficiently integrating services across components of the model and continuum of care and directly addressing SDOH, the TMM offers a powerful approach to address the needs of rural communities who may lack local health services and experience structural inequities in health. It also aligns with and advances the goals and priorities of health equity frameworks that call attention to local contexts, social and structural determinants of health, and the need for coordinated approaches to improve the health of populations [29]. To that end, we identified three characteristics of the TMM that make it a particularly promising care delivery model for underserved rural populations which includes, (a) reach; (b) bridge to care; and (c) scalability. These characteristics are also what make the TMM both flexible and adaptable, such that it can be tailored to each unique rural community.

#### 1.3.1 Reach

The TMM has the potential to expand the reach of existing care delivery models, including MHCs and SM, by traveling to geographically distant communities and difficult‐to‐reach locations, meeting people where they are. Using mobile units and smaller vehicles, providers have the ability to bring needed services to rural communities with mobile units, as well as to more isolated individuals or communities in rural or remote locations with tailgate teams.

#### 1.3.2 Bridge to Care

The TMM provides multiple entry points to care, which facilitates connections to local resources and pathways for integrating individuals into existing health systems. While the scope and level of services available from tailgate teams, mobile units, and the central medical center hubs differ, the goal of this care delivery model is to create bridges from one level of care to the next as needed by the people served. For example, the TMM expands the provision of care by combining the ‘easy trust' of tailgate teams—approachability and delivery of care where people are—with easier access to medical equipment and services provided by the mobile units. The overarching goal is to develop trusting relationships and provide access to additional services (e.g., specialty care) at a brick‐and‐mortar clinic. Connecting individuals to other resources (e.g., food or housing services) and taking steps to secure insurance coverage are also means of integrating existing services.

#### 1.3.3 Scalability

Expanding services to additional populations and communities requires the ability to scale operations. Although scalability is a complex and multifaceted process, several features of the TMM facilitate scaling of operations. The TMM combines small tailgate teams with the power of mobile units, and the support of central medical hubs to deliver services more efficiently in rural areas. Mobile units usually have multiple patient rooms, increasing the efficiency of care delivery in the communities served. However, mobile units, which require some customization, can have high upfront costs and be costly to maintain. Conversely, tailgate teams require less upfront capital to launch and expand through additional teams, as well as lower continuous capital to sustain operations. Moreover, specialty vehicles or modifications are not needed to deploy tailgate teams. Therefore, existing infrastructure, such as personal or fleet vehicles, can be used to quickly scale operations and provide access to additional communities that may be geographically separated. Point of care diagnostics, web‐based electronic health records, and other resources can be shared across components of the TMM, reducing the cost of deploying both mobile units and tailgate teams. The integrated nature and the multi‐level hub‐and‐spoke design of the TMM are features that can lower operating costs and facilitate expansion and scalability to meet local needs.

Despite the flexibility and adaptability of the TMM, it may not be the most efficient or effective approach for all communities or settings. For example, tailgate teams may not be the most efficient means for care delivery in rural or remote areas due to the significant time for travel between individuals.

Workforce challenges, such as limited providers or staff to support the operations of mobile units or tailgate teams, may also influence implementation and scalability of the TMM. In regions with highly fragmented health care systems, the inability to share resources or information across data systems may limit the reach and impact of the TMM. To identify context‐specific barriers and facilitators, assess priority needs, understand community readiness and facilitate local engagement, implementing and scaling the TMM should be guided by models or frameworks, such as the Precede‐Proceed Model or the RE‐AIM Framework, (Fogarty International Center, 2023). These frameworks and models integrate program evaluation, which includes impact assessment Center [30].

## 2 Limitations

It is important to note that the proposed TMM remains a largely untested model for health care delivery. Therefore, thoughtful planning and implementation is needed to tailor the TMM for local settings. While treatment advances, such as point of care testing, have been integrated from existing care delivery models (e.g., MHC and SM) with life‐saving care, like hemorrhage control, and approaches used by the military (e.g., tailgate teams), much remains unknown in terms of understanding the feasibility, sustainability, and overall impact of the TMM. Given the significant potential of the TMM, we believe further investigation and research is justified. For example, geography, road conditions, and weather will influence access and care delivery (e.g., mobile unit or tailgate team) and impact overall efficiency and impact. There are also potential challenges with the implementation, financing, and scalability of this model that warrant exploration. While the TMM can have lower start‐up costs than other care delivery models, some investment in supplies and equipment may be needed. Moreover, scaling operations will require financial capital. Local needs, resources, and systems will influence the cost of direct services as well as how those costs will be covered (e.g., free, out of pocket, reimbursed). In situations where direct care services are reimbursed (i.e., billed to insurance), rates may not be sufficient to support lower volume of patients, depending upon the services delivered. Given the significant potential of the TMM to address many of the structural factors that perpetuate health inequities in rural communities by better addressing the unique health needs in rural and remote locations and creating bridges to existing health resources and social services, we believe further development of the TMM and research of its impacts is warranted.

## 3 Conclusion

Achieving health equity is a public health priority, and one that should be approach with urgency for rural and underserved populations. Addressing health inequities in rural and remote areas necessitates the development of care delivery models that directly confront the social, environmental, economic, and cultural factors creating structural barriers to optimal health within local communities. To that end, we have conceptualized and proposed the TMM—a novel, integrative model for rural, community‐based health care delivery that strategically leverages the strengths of both MHCs and SM, enhanced by the flexibility, adaptability, and responsiveness of the US military's tailgate medicine approach. Although this model remains untested in the community setting, we argue that the TMM is an innovative and feasible care delivery model that can advance health equity in rural communities through expanded reach of services, bridges to health promoting resources and care, and scalability of operations.

## Author Contributions

**Michael McShane:** conceptualization (lead), writing – original draft preparation (lead), writing –review and editing (equal). **Jennifer Kowalkowski:** conceptualization (equal), writing – original draft (equal), writing – review and editing (equal), visualization (lead). **Kristina Brant:** conceptualization (equal), writing – original draft preparation (equal), writing – review and editing (equal). **Joel Segel:** writing – original draft preparation (equal), writing – review and editing (supporting). **Ashely Visco:** writing – original draft (equal), writing – review and editing (supporting). **Phillip Volpe:** conceptualization (supporting), writing – original draft preparation (supporting), writing – review and editing (supporting). **Mark Stephens:** conceptualization (equal), writing – original draft preparation (supporting), writing – review and editing (supporting).

## Conflicts of Interest

The authors declare no conflicts of interest.

## Transparency Statement

The lead author Michael P. McShane affirms that this manuscript is an honest, accurate, and transparent account of the study being reported; that no important aspects of the study have been omitted; and that any discrepancies from the study as planned (and, if relevant, registered) have been explained.

## Data Availability

The authors have nothing to report.
